# Integrative Analysis Uncovers *SETD5* as an Epigenetic Regulator of Transcriptional and Immune Tumor Programs Across Human Cancers

**DOI:** 10.3390/cimb48070742

**Published:** 2026-07-21

**Authors:** Ana Cristina Moura Gualberto, Brunna Letícia de Oliveira Santana, Mariana Braccialli de Loyola, Yasmim Sampaio da Costa, Fábio Pittella-Silva

**Affiliations:** 1 Laboratory of Molecular Pathology of Cancer, Faculty of Health Sciences, University of Brasilia, Brasilia 70910-900, Brazil; ana.gualberto@unb.br (A.C.M.G.); brunna.los@hotmail.com (B.L.d.O.S.); marianabraccialli@outlook.com (M.B.d.L.); 2 Laboratory on Thymus Research, Oswaldo Cruz Institute, Oswaldo Cruz Foundation, Rio de Janeiro 21040-900, Brazil; yasmimsampa2@hotmail.com; 3 National Institute of Science and Technology on Neuroimmunomodulation, Oswaldo Cruz Institute, Oswaldo Cruz Foundation, Rio de Janeiro 21040-900, Brazil

**Keywords:** *SETD5*, pan-cancer, epigenetic regulation, tumor microenvironment, immune regulation

## Abstract

*SETD5* (SET domain-containing 5) is a chromatin-associated regulator increasingly recognized as dysregulated in human malignancies; however, its contribution to tumor biology and tumor–immune interactions remain undefined. We performed an integrative pan-cancer multi-omics analysis to define the landscape of *SETD5* dysregulation across cancer types. Transcriptomic, genomic, and epigenetic datasets were integrated to evaluate *SETD5* alterations and molecular associations. Protein interaction and pathway enrichment analyses were conducted using STRING, GO, and KEGG, and immunogenomic profiling was used to interrogate associations between *SETD5* expression, immune infiltration, and checkpoint programs. *SETD5* was overexpressed across multiple malignancies, with low mutation frequency but recurrent copy-number gains. Promoter hypomethylation was detected in a subset of tumors with increased *SETD5* expression, suggesting a possible association with epigenetic regulation. Pathway analyses linked *SETD5* to macromolecule methylation and transcriptional regulation. *SETD5* expression correlated positively with infiltration of macrophages, neutrophils, and dendritic cells, whereas associations with CD8^+^ and CD4^+^ T cells varied by tumor type. Tumor type-specific correlations were observed between *SETD5* and immune checkpoints genes, including *PD-L1* and *TIM-3*, suggesting an association with immunoregulatory tumor states. These findings identify S*ETD5* as a recurrently deregulated epigenetic regulator and highlight its potential role in transcriptional control and tumor immune modulation.

## 1. Introduction

Epigenetic reprogramming is a hallmark of cancer, reshaping the transcriptional networks that sustain malignant characteristics. Alterations in chromatin-regulatory mechanisms can lead to significant changes in gene expression and hallmark features of tumor progression including uncontrolled proliferation, inactivation of suppressor genes, activation of oncogenes and immune escape [1,2]. Immune evasion has emerged as a central determinant of tumor persistence and treatment failure and is increasingly recognized as being influenced by epigenetic mechanisms operating both within cancer cells and in the surrounding microenvironment [3,4]. Chromatin modifiers, including lysine methyltransferases (KMTs), govern transcriptional states that shape cellular behavior, and increasing evidence suggests that their dysregulation contributes to the establishment of tumor-permissive immune landscapes [5].

Among KMTs, *SETD5* (SET domain-containing protein 5) remains one of the least characterized, despite growing evidence implicating it in cancer-associated regulatory processes. Integrative analyses of transcriptomic datasets from patient cohorts, together with functional siRNA (small interfering RNA) and CRISPR (Clustered Regularly Interspaced Short Palindromic Repeats)-based screening studies, have identified *SETD5* as a contributor to transcriptional programs associated with tumor progression. Elevated expression has been reported for multiple cancer types and is associated with adverse clinical features [6,7,8,9,10]. Notably, recent functional studies have demonstrated that *SETD5* regulates glycolysis through the EP300/HIF-1α axis in breast cancer stem-like cells [9], promotes adaptive drug resistance in pancreatic cancer by scaffolding a co-repressor complex containing HDAC3 and G9a [10], and facilitates cancer stemness while repressing ferroptosis via m6A-mediated PKM2 stabilization in non-small cell lung cancer [11]. In addition, the structural similarity of *SETD5* to other SET-domain methyltransferases, in conjunction with evidence of its integration into networks regulating chromatin, supports the hypothesis that *SETD5* functions as an epigenetic modulator of oncogenic gene expression [12].

Epigenetic mechanisms, including promoter methylation, have been associated with changes in *SETD5* expression [12]. Furthermore, non-coding RNAs, including microRNAs and long non-coding RNAs, have been postulated as post-transcriptional modulators of *SETD5*, thereby associating it with more extensive regulatory circuits involving RNA-mediated chromatin control [13,14]. These interdependent regulatory layers may collectively shape chromatin accessibility, transcriptional fidelity, and cellular adaptability. This suggests that dynamic control of *SETD5* expression could contribute to tumor evolution.

Therefore, a comprehensive understanding of *SETD5′s* role in cancer is crucial for identifying epigenetic vulnerabilities that may be therapeutically actionable. Elucidation of the regulatory networks and functional dependencies in which *SETD5* operates has the potential to reveal novel opportunities to target transcriptional programs that support malignancy. In consideration of the emerging evidence that indicates a correlation between chromatin modifiers and immune landscape remodeling, it is essential to investigate the relationship between *SETD5* and tumor immune contexture. This investigation may yield critical insights into the mechanisms of immune evasion and therapeutic resistance. In this study, we investigated the role of *SETD5* across multiple tumor types through a comprehensive in silico analysis encompassing expression profiles, genomic alterations, promoter methylation, protein interaction networks, and functional associations. This work provides a foundation for future research by examining *SETD5* within these multidimensional datasets and by exploring the potential of *SETD5* as an epigenetic regulator of tumor progression and immune modulation.

## 2. Materials and Methods

### 2.1. Database Analysis

The TIMER database (https://compbio.cn/timer2/, accessed on 7 May 2026) was used to analyze the expression of *SETD5* across various tumor types, which provides RNA sequencing (RNA-seq) data derived from The Cancer Genome Atlas. The gene expression levels were assessed using the “Gene_DE” module and boxplots comparing tumor and adjacent normal tissues were obtained from the platform. The UALCAN database (http://ualcan.path.uab.edu, accessed on 20 May 2026) was employed to evaluate *SETD5* expression in tumor tissues compared to normal tissues in the cancer types selected from TIMER. Statistical comparisons of *SETD5* expression between tumor and adjacent normal tissues were performed internally by each platform (unpaired Student’s *t*-test for UALCAN; Wilcoxon rank-sum test for TIMER), with significance set at *p* < 0.05 for each cancer type independently. Data on mutation frequency and copy number alterations (CNAs) were obtained from the cBioPortal for Cancer Genomics (www.cbioportal.org, accessed on 22 May 2026). CNA data were generated using the GISTIC (Genomic Identification of Significant Targets in Cancer) algorithm, where a value of −2 indicates a homozygous deletion and a value of 2 indicates gene amplification.

### 2.2. Data Download

RNA-seq data from primary tumor samples were obtained from The Cancer Genome Atlas (TCGA) using the ‘TCGAbiolinks’ package in R (v.4.5.3). Raw counts were normalized to transcripts per million (TPM) and log2-transformed [log2(TPM + 1)]. Duplicate patient samples were removed prior to analysis. The numbers of tumor and normal samples included for each cancer type are summarized in Supplementary Tables S1–S5.

### 2.3. Promoter Methylation Levels

We analyzed the DNA methylation levels of the *SETD5* promoter across multiple cancer types using publicly available data from TCGA). Methylation profiling was performed using the Illumina HumanMethylation450 BeadChip platform (Illumina Inc., San Diego, CA, USA),focusing specifically on the promoter-associated probe cg01564818, located within the promoter region of *SETD5*. Beta values were compared between primary tumor samples and normal tissues across a range of tumor types.

Methylation data were downloaded from the TCGA data portal and processed using R software version 4.3.1 (R Foundation for Statistical Computing, Vienna, Austria) for initial handling and quality control. Further data organization was conducted in Microsoft Excel. GraphPad Prism (8.0.2; GraphPad Software, San Diego, CA, USA) was used for statistical analyses and graphical representations. Differences in methylation levels between tumor and normal samples were assessed using either unpaired Student’s *t*-tests or Mann–Whitney U tests according to data distribution with significance set at *p* < 0.05.

### 2.4. Protein–Protein Interaction Network

A protein–protein interaction (PPI) network was constructed using the STRING database (version 12.0, https://string-db.org, accessed on 22 May 2026). In this network, nodes represent proteins, and edges represent a potential functional association between each protein based on multiple types of evidence, including curated databases, experimental data, gene neighborhood, gene fusions, co-occurrence, text mining, co-expression, and protein homology. A confidence score threshold of 0.4 (medium confidence) was applied. STRING interaction scores, which range from 0 to 1, were used to assess the confidence level of the predicted functional associations.

### 2.5. GO and KEGG Pathway Enrichment Analyses

To investigate the biological functions associated with *SETD5*-related genes, we performed a functional enrichment analysis using the Gene Ontology (GO) ontologies and the Kyoto Encyclopedia of Genes and Genomes (KEGG) pathway database. The selected genes were submitted to the enrichGO (for biological processes—BP) and enrichKEGG functions of the clusterProfiler package (v.4.x) in the R environment (R v.4.5.3; RStudio 2025.09.0+387) [15]. Terms with an adjusted FDR value of less than 0.05 were considered significantly enriched.

### 2.6. Infiltration Analysis of Immune Cells

Tumor purity was estimated using the ESTIMATE algorithm. Immune cell infiltration levels were inferred from TPM-normalized expression data using the TIMER algorithm implemented in the ‘immunedeconv’ package (v.2.1.0) in R (v.4.5.3).

### 2.7. Correlation Analysis Between SETD5 and Immune Cells

The association between *SETD5* expression and immune cell infiltration was assessed using partial Spearman correlation analysis adjusted for tumor purity with the ppcor package (v.1.1). *p*-values were corrected for multiple testing using the Benjamini–Hochberg method, and adjusted *p*-values < 0.05 were considered statistically significant. Correlation heatmaps were generated using the ComplexHeatmap package (v.2.26.1). Non-significant correlations were represented as grey cells.

### 2.8. Correlation Analysis Between SETD5 and Immune Genes

Spearman correlation analysis was performed to evaluate the association between *SETD5* and immune checkpoint genes (*PDCD1*, *CD274*, *CTLA4*, *LAG3*, and *HAVCR2*). Correlation matrices were visualized using the corrplot package (v.0.95).

### 2.9. Survival Analysis

Overall survival analysis was performed using clinical and transcriptomic data obtained using ‘TCGAbiolinks’ package in R (v.4.5.3). Patients were stratified into high- and low-expression groups according to the median *SETD5* expression within each tumor cohort. Patients without death events were treated as censored observations. Survival analyses and graphical representations were performed in R using the packages survival package (v.3.8-6) and survminer package (v.0.5.2). Confidence intervals of 95% were displayed for all Kaplan–Meier curves, and risk tables indicating the number of patients at risk over time were included.

## 3. Results

### 3.1. SETD5 Is Widely Upregulated Across Multiple Human Cancers

*SETD5* mRNA expression was evaluated between tumor and normal tissue from TIMER database. This global analysis showed upregulation of *SETD5* in bladder urothelial carcinoma (BLCA), colon adenocarcinoma (COAD), esophageal carcinoma (ESCA), head and neck squamous cell carcinoma (HNSC), liver hepatocellular carcinoma (LIHC), lung adenocarcinoma (LUAD), lung squamous cell carcinoma (LUSC), rectum adenocarcinoma (READ) and stomach adenocarcinoma (STAD) (Supplementary Figure S1).

**Figure 1:**
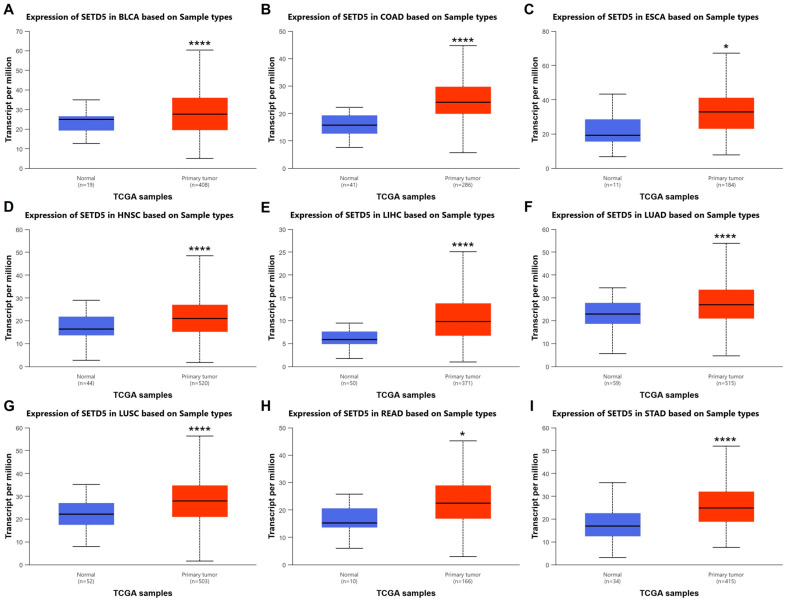
*SETD5* expression in tumor and normal tissue. (**A**) Bladder urothelial carcinoma—BLCA. (**B**) Colon adenocarcinoma—COAD. (**C**) Esophageal carcinoma—ESCA. (**D**) Head and neck squamous cell carcinoma—HNSC. (**E**) Liver hepatocellular carcinoma—LIHC. (**F**) Lung adenocarcinoma—LUAD. (**G**) Lung squamous cell carcinoma—LUSC. (**H**) Rectum adenocarcinoma—READ. (**I**) Stomach adenocarcinoma—STAD. Unpaired *T*-test (* *p* < 0.05 and **** *p* < 0.0001).

Subsequently, an individual analysis of these tumors was performed using the UALCAN database to confirm *SETD5* expression differences in tumor and normal tissues. *SETD5* expression was significantly higher in the following tumor types when compared to normal tissue: BLCA (*p* = 2.20 × 10^−5^), COAD (*p* = 1.62 × 10^−12^), ESCA (*p* = 2.32 × 10^−2^), HNSC (*p* = 5.10 × 10^−7^), LIHC (*p* < 1 × 10^−12^), LUAD (*p* = 1.28 × 10^−9^), LUSC (*p* = 8.81 × 10^−11^), READ (*p* = 2.37 × 10^−2^) and STAD (*p* = 4.12 × 10^−9^) (Figure 1A–I).

### 3.2. Frequency of Mutations and Copy Number Alterations

**Figure 2:**
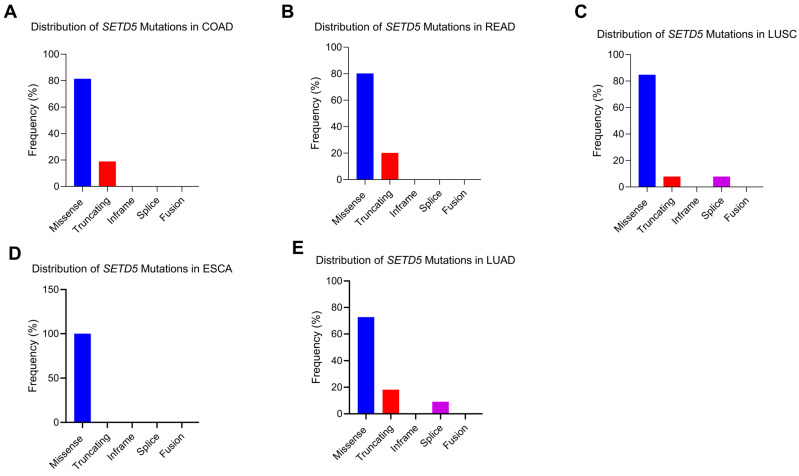
Frequencies of mutation types (**A**) Colon adenocarcinoma (COAD). (**B**) Rectum adenocarcinoma (READ). (**C**) Lung squamous cell carcinoma (LUSC). (**D**) Esophageal carcinoma (ESCA). (**E**) Lung adenocarcinoma (LUAD).

We used cohorts from the cBio Portal to analyze the frequency of mutations and copy number alterations (CNAs) (Table 1). The mutation frequency of *SETD5* was generally low. However, colon adenocarcinoma (COAD), rectum adenocarcinoma (READ), lung adenocarcinoma (LUAD), lung squamous cell carcinoma (LUSC), liver hepatocellular carcinoma (LIHC) and esophageal carcinoma (ESCA) showed frequencies above 1.5%. Among these tumors, somatic *SETD5* variants were detected in 3.9% of COAD cases and 4.7% of READ cases. Interestingly, both tumor types exhibited approximately 80% missense mutations and 20% truncating mutations. In LUSC, the mutation rate was 5.9%, with ~80% missense and ~10% truncating and splice mutations. In ESCA, *SETD5* variants were found in 2% of cases, all of which were missense mutations (Figure 2A–E).

Regarding CNAs, the overall frequency of alterations was relatively low. Nevertheless, the highest amplification frequencies were observed in esophageal carcinoma (ESCA), and bladder urothelial carcinoma (BLCA): 2.6% and 7.5%, respectively (Table 1).

### 3.3. Promoter Methylation Levels in Cancer Tissue and Normal Tissue

To investigate the epigenetic regulation of *SETD5* across different cancer types, we analyzed promoter methylation data from The Cancer Genome Atlas (TCGA). DNA methylation profiles were obtained using the Illumina HumanMethylation450 BeadChip platform, and the analysis focused on the promoter-associated probe cg01564818, located in the promoter region of *SETD5*. We compared methylation levels between primary tumor samples and normal tissues across multiple tumor types.

**Figure 3:**
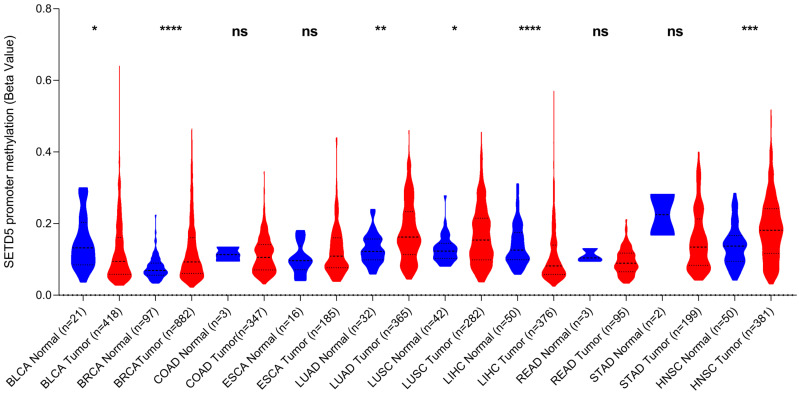
*SETD5* promoter methylation differences between tumor and normal tissues in TCGA datasets. Significant hypomethylation in tumors was observed in BLCA and LIHC (* *p* < 0.05; **** *p* < 0.0001), while HNSC showed tumor-specific hypermethylation (*** *p* < 0.001). LUAD and LUSC also exhibited higher methylation in tumor tissues (** *p* < 0.01; * *p* < 0.05). Statistical significance was determined using or Mann–Whitney U test. Dotted lines indicate the median value of each group.

Significant differences in methylation patterns in the promoter region of *SETD5* were identified in several cancers. In BLCA and LIHC, we observed significantly lower methylation levels in tumor samples compared to normal tissues (*p* < 0.05 and *p* < 0.0001, respectively, Figure 3). On the other hand, higher methylation levels in tumors were detected in HNSC, LUAD and LUSC (*p* < 0.001, *p* < 0.01 and *p* < 0.05, respectively). Although READ and colon adenocarcinoma COAD appeared to follow a similar trend of decreased methylation in tumor samples, these differences were not statistically significant, potentially due to the limited number of available normal samples in these cohorts.

### 3.4. SETD5 Interaction Network Indicates a Role in Chromatin Remodeling and Transcriptional Regulation

**Figure 4:**
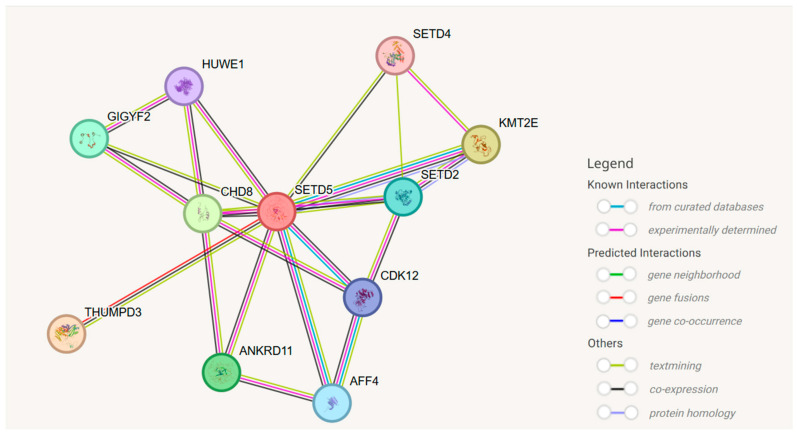
Protein–protein interaction network built in STRING (version 12.0), considering interactions with a score ≥ 0.4 (medium confidence). The nodes represent proteins encoded by the genes of interest, and the edges represent interactions known (from curated databases and experimental evidence) or predicted (by genomic neighborhood, gene fusions, co-occurrence, co-expression, text mining and homology).

To explore the potential functional partners of SETD5, a protein–protein interaction (PPI) network was generated using the STRING database. The analysis included both known and predicted interactions (Figure 4). The network interaction scores were used to evaluate the confidence of predicted functional partners of SETD5. Among these, the ten highest-confidence interacting partners were selected for downstream functional analysis (Supplementary Table S6). The lysine methyltransferases KMT2E (0.702), SETD2 (0.599) and SETD4 (0.576) showed high and moderate interaction confidence (Supplementary Table S6). These findings suggest that SETD5 may participate in chromatin remodeling and transcriptional regulation through its interaction with these partners. Other partners, such as THUMPD3 (interaction score 0.732), were also retrieved; THUMPD3 is a tRNA-modifying enzyme that has additionally been implicated in alternative splicing regulation and lung cancer proliferation.

### 3.5. Analysis of Functional Enrichment

**Figure 5:**
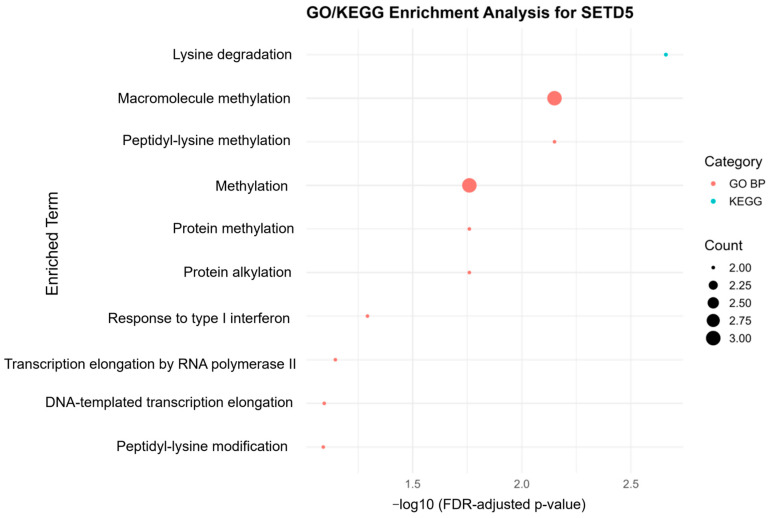
GO/KEGG pathway enrichment analysis of *SETD5*-related genes. The dot plot graph shows the main terms enriched in the Gene Ontology biological processes (GO BP, in red) and KEGG pathways (in blue) categories. The size of the dots indicates the number of genes associated with each pathway (Terms with an adjusted FDR below 0.05 were included).

To explore the functional roles of the ten *SETD5*-associated genes identified through STRING analysis, we performed an enrichment analysis using Gene Ontology (GO) and KEGG pathways. The analysis showed an enrichment in biological pathways related to macromolecule methylation (GO:0043414), protein methylation (GO:0006479), and peptidyl-lysine methylation (GO:0018022), which indicates an association with epigenetic regulatory mechanisms. An enrichment in pathways related to transcriptional control was also observed, such as “transcription elongation by RNA polymerase II” (GO:0006368) and “DNA-templated transcription elongation” (GO:0006354). Notably, the KEGG pathway “Lysine degradation” (hsa00310) was enriched, highlighting a potential link between *SETD5*-associated genes and lysine metabolism (Figure 5). Taken together, these findings highlight the role of *SETD5* as an epigenetic modifier. This data also suggests that *SETD5* influences the process of gene expression at the transcriptional level.

### 3.6. SETD5 Correlates with Immune Infiltration Across Tumors

**Figure 6:**
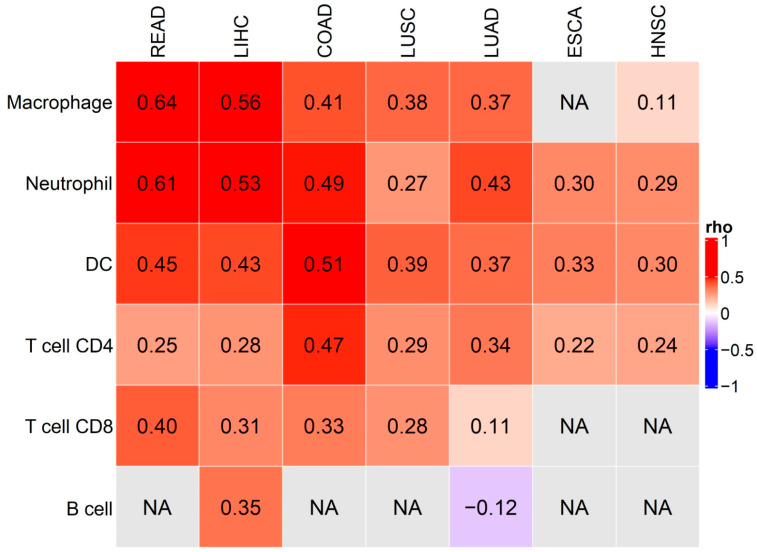
Correlation analysis between *SETD5* expression and immune cell infiltration across TCGA tumor types. Heatmap colors represent correlation coefficients (ρ), ranging from negative (blue) to positive (red) correlations. Gray cells (NA) indicates that no statistically significant correlation was identified in the corresponding analysis.

To assess the potential association between *SETD5* expression and remodeling of the tumor immune microenvironment, we quantified its correlation with key immune cell populations across seven tumor types (Figure 6). *SETD5* expression showed variable, tumor type-dependent positive correlations with multiple immune cell populations across the seven tumor cohorts analyzed, highlighting a context-specific relationship between *SETD5* and immune infiltration patterns. The strongest and most consistent associations were observed for macrophages (ρ = 0.37–0.64), with the highest values in READ (ρ = 0.64) and LIHC (ρ = 0.56). Notably, *SETD5* showed no detectable macrophage correlation in ESCA samples (NA). Neutrophils exhibited robust correlations (ρ = 0.27–0.61), with the highest values in READ (ρ = 0.61) and LIHC (ρ = 0.53), were among the strongest associations observed across all cell types. Dendritic cells showed moderate and consistent correlations across cohorts (ρ = 0.30–0.51), with the highest value in COAD (ρ = 0.51). CD4^+^ T cell correlations were modest (ρ = 0.22–0.47), with the strongest association in COAD (ρ = 0.47). CD8^+^ T cell correlations ranged from ρ = 0.11 in LUAD to ρ = 0.40 in READ, with no detectable association in ESCA and HNSC (NA). B cells showed detectable correlations only in LIHC (ρ = 0.35) and a slight negative correlation in LUAD (ρ = −0.12), with non-significant (NA) values in the remaining cohorts. Collectively, these findings indicate that *SETD5* expression is most consistently associated with innate immune cell populations such as macrophages, neutrophils, and dendritic cells rather than cytotoxic lymphocytes, suggesting a preferential link between *SETD5* and innate immune microenvironment remodeling across cancer types.

### 3.7. SETD5 Expression Is Associated with Immune Checkpoint Signatures Across Tumor Types

**Figure 7:**
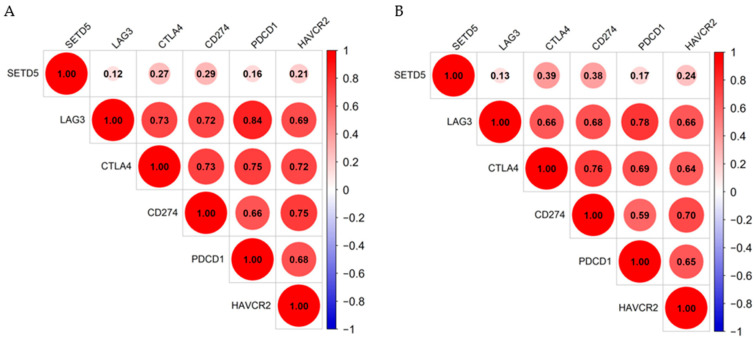
Correlation analysis between *SETD5* and immune checkpoint genes across TCGA tumor samples. (**A**) COAD. (**B**) READ. Spearman correlation coefficients (ρ) are represented by color intensity, ranging from negative (blue) to positive (red) correlations. Only pairwise complete observations were included in the analysis.

To determine whether *SETD5* expression is associated with T-cell inhibitory networks, correlation analyses were performed between *SETD5* and key immune checkpoint genes across six tumor types. Among these, COAD and READ exhibited the most consistent and pronounced correlation patterns and were therefore selected for presentation in the main figure (Figure 7).

Tumor type-specific correlations between *SETD5* and immune checkpoint genes were observed. In COAD and READ, *SETD5* showed modest positive correlations with all evaluated immune checkpoint genes, with the highest values observed for *CD274* (*PD-L1*; ρ = 0.29 and ρ = 0.38, respectively) and *CTLA4* (ρ = 0.27 and ρ = 0.39, respectively). Correlations with *HAVCR2* (*TIM-3*; ρ = 0.21 and ρ = 0.24) and *PDCD1* (*PD-1*; ρ = 0.16 and ρ = 0.17) were comparatively weaker. Notably, among the checkpoint molecules themselves, strong inter-checkpoint correlations were observed, particularly between *LAG3* and *PDCD1* (ρ = 0.84 in COAD; ρ = 0.78 in READ), and between *CTLA4* and *PDCD1* (ρ = 0.75 in COAD; ρ = 0.69 in READ), suggesting coordinated checkpoint co-expression in these tumor microenvironments. In contrast, LUSC, *SETD5* showed near-zero or slightly negative correlations with *CTLA4* (ρ = −0.03), *PDCD1* (ρ = −0.02), and *HAVCR2* (ρ = −0.08), indicating tumor-type specificity in the *SETD5*–checkpoint relationship. Correlation analyses for the remaining four tumor types are provided in Supplementary Figure S2A–D.

### 3.8. SETD5 Expression Shows Tumor-Specific Associations with Overall Survival

Survival analyses were performed to investigate the prognostic relevance of *SETD5* expression across tumor types. Although most cancers did not exhibit statistically significant associations between *SETD5* expression and overall survival, tumor-specific effects were observed in selected cohorts (Supplementary Figure S3). In LIHC, elevated *SETD5* expression was associated with worse overall survival (HR = 1.61, 95% CI: 1.13–2.28, *p* = 0.073). In contrast, in READ, higher SETD5 expression correlated with improved survival outcomes (HR = 0.42, 95% CI: 0.19–0.95, *p* = 0.031). These findings suggest that the prognostic impact of *SETD5* may be context-dependent and influenced by tumor-specific biological programs.

## 4. Discussion

The present study suggests that *SETD5* is recurrently dysregulated across multiple cancer types through coordinated transcriptional, genetic, and epigenetic mechanisms. Our pan-cancer analysis reveals consistent upregulation in nine distinct malignancies, accompanied by tumor-specific patterns of promoter methylation and copy-number alterations. Beyond these genomic and epigenomic features, our findings further demonstrate that elevated *SETD5* expression is associated with immune-related transcription programs, including coordinated expression of multiple immune checkpoint genes across tumor types. Collectively, these findings support the hypothesis that *SETD5* acts as a candidate epigenetic regulator of oncogenic and immunomodulatory programs and suggest a potential role in cancer-associated transcriptional reprogramming that extends to the shaping of the tumor immune microenvironment.

The observed upregulation of *SETD5* in BLCA, COAD, ESCA, HNSC, LUSC, and LUAD aligns with evidence from large-scale transcriptomic profiling and functional genomic screens that have recurrently identified epigenetic regulators as dysregulated across multiple tumor types [16,17,18]. While SET domain-containing proteins have been extensively characterized as key regulators of chromatin states, *SETD5* has remained comparatively understudied despite its structural similarity to established histone methyltransferases. These findings indicate that *SETD5* expression is elevated independently of high mutation burden, suggesting that genetic alterations are not the primary driver of its deregulation in most tumor contexts [18]. The consistent upregulation of *SETD5* across multiple cancer types, coupled with its integration into chromatin regulatory networks, suggests potential vulnerabilities that may be therapeutically exploited. The development of selective small-molecule inhibitors targeting SET domain methyltransferases has shown promise in preclinical models [19,20].

The mutation frequencies observed in colorectal (COAD: 3.9%; READ: 4.7%) and lung squamous cell carcinoma (5.9%) are consistent with the hypermutated phenotypes characteristic of these malignancies [21,22]. However, the predominance of missense over truncating mutations suggests selective pressure to maintain *SETD5* catalytic function rather than loss-of-function. Copy-number analysis revealed appreciable amplification frequencies in esophageal carcinoma (ESCA; 2.6%) and bladder urothelial carcinoma (BLCA; 7.5%). These findings are consistent with the well-established role of chromosomal instability in driving recurrent copy-number alteration in cancer that *SETD5* amplification occurs in a subset of ESCA and BLCA tumors [23,24].

Our methylation profiling showed tumor-type specificity in *SETD5* promoter methylation, with hypomethylation observed in BLCA and LIHC correlating inversely with elevated expression. This result is consistent with previous observations that promoter hypomethylation may contribute to transcriptional activation [5,25]. In contrast, the analysis of HNSC, LUAD and LUSC revealed a pattern of hypermethylation accompanied by elevated *SETD5* expression. This finding suggests the potential involvement of alternative regulatory mechanisms, such as enhancer activation, chromatin remodeling, or transcription factor binding, which may supersede the effects of methylation-mediated repression in these specific contexts [26]. The absence of statistical significance in COAD and READ, despite discernible trends toward hypomethylation, may be indicative of limited representation of normal tissue in the TCGA cohorts. These observations are based on a promoter-associated probe, and future studies evaluating additional promoter sites across the *SETD5* locus may provide a more comprehensive characterization of its methylation profile.

Protein interaction network analysis based on STRING identified THUMPD3, KMT2E (MLL5), SETD2 and SETD4 as predicted functional partners of SETD5, with interaction scores of 0.732, 0.702, 0.599 and 0.576, respectively. Despite the absence of direct biochemical validation of these interactions, the association of SETD5 with chromatin regulators that share the SET-domain architecture, or the function of chromatin remodeling suggests the potential for these proteins to participate in shared, chromatin-associated regulatory assemblies. It has been noted that *KMT2E*, a member of the MLL family of H3K4 methyltransferases, is frequently mutated in microsatellite instability–high colorectal tumors. This feature has been associated with reduced gene expression [27]. Similarly, *SETD2*, a well-established tumor suppressor, exhibits a high mutation frequency in colorectal cancer, consistent with loss-of-function events [28]. *SETD4* is overexpressed in several tumor types, and its epigenetic mechanism is associated with tumor progression [29]. Taken together, these observations suggest that, in network-based analysis, chromatin regulators that are positively associated with *SETD5* may be functionally compromised in colorectal tumors, potentially influencing chromatin-dependent transcriptional regulation in this context.

THUMPD3 is a methyltransferase that forms a complex with TRMT112 to catalyze N^2^-methylguanosine (m^2^G) formation at the sixth or seventh guanine position of tRNA [30,31]. The association between SETD5 and THUMPD3 may be related to their proximity on chromosome 3p25.3 [32]. Notably, SETD5-AS1, also known as THUMPD3-AS1 according to NCBI Gene, is an antisense transcript implicated in the regulation of epigenetic modifications [33]. Therefore, we hypothesize that the high interaction scores between SETD5 and THUMPD3 may be mediated by this overlapping antisense lncRNA, which could regulate the expression of both genes. Additionally, a recent study showed that ANKRD11 (0.669) has been described as a transcriptional regulator of *SETD5*, being responsible for recruiting WDR5 and increasing H3K4me3 levels at the *SETD5* promoter region [34].

Gene Ontology and KEGG pathway enrichment analyses demonstrated significant representation of *SETD5*-associated genes in macromolecule methylation (GO:0043414), protein methylation (GO:0006479), and peptidyl-lysine methylation (GO:0018022), consistent with its predicted role as a chromatin-associated epigenetic regulator [35,36]. Enrichment in transcription elongation by RNA polymerase II (GO:0006368) and DNA-templated transcription elongation (GO:0006354) further supports the hypothesis that *SETD5* functions within the transcriptional machinery to modulate gene expression kinetics [37,38]. The enrichment of the KEGG pathway “Lysine degradation” (hsa00310) is a subject of particular interest, as lysine metabolism is increasingly recognized as a metabolic vulnerability in cancer, with therapeutic implications for targeting epigenetic regulators [39,40]. *CDK12* was strongly correlated with *SETD5* (0.590) and regulates transcription through RNA polymerase II phosphorylation, as indicated by our KEGG analysis. *CDK12* is overexpressed in various cancer types and contributes to oncogenesis by promoting the WNT/β-catenin and MAPK pathways. In addition, it is associated with modulation of the tumor microenvironment, facilitating immune evasion through the upregulation of *PD-1* and *CTLA-4* and the recruitment of immunosuppressive cells [41].

An emerging dimension of *SETD5* biology pertains to its role in metabolic reprogramming. In breast cancer, Yang et al. demonstrated that *SETD5* is enriched in cancer stem-like cells and promotes glycolysis through the EP300/HIF-1α axis, with *SETD5* knockdown significantly reducing hexokinase-2 and *PFKFB3* expression under hypoxic conditions [9]. Similarly, Shi et al. showed that *SETD5* silencing in gastric cancer cells inhibited glycolysis and tumor growth through downregulation of the AKT signaling pathway [42]. Most recently, Liu et al. (2025) demonstrated that *SETD5* functions as an active H3K36me3 methyltransferase in non-small cell lung cancer, where it facilitates stemness and represses ferroptosis through m6A-mediated *PKM2* stabilization involving *METTL14* and *YTHDF1* [11]. These findings are particularly relevant to our observation of *SETD5* upregulation in LUAD, LUSC, and STAD, as they suggest that elevated *SETD5* expression may confer metabolic advantages to tumor cells beyond the transcriptional regulatory functions identified in our enrichment analyses. The convergence of epigenetic regulation, metabolic adaptation, and cancer stemness maintenance through *SETD5* underscores its potential as a multifaceted therapeutic target.

Importantly, the molecular mechanism by which *SETD5* exerts its regulatory functions may not be limited to canonical histone methyltransferase activity. Wang et al. (2020) demonstrated in pancreatic ductal adenocarcinoma that *SETD5* lacks direct histone methyltransferase activity but instead scaffolds a co-repressor complex containing HDAC3 and G9a, thereby coupling selective H3K9 deacetylation with methylation at target gene promoters [10]. This scaffolding function directed the silencing of drug resistance-associated genes and the pharmacological co-targeting of MEK1/2, HDAC3, and G9a sustained tumor growth inhibition in vivo. Furthermore, Park et al. (2022) showed in hepatocellular carcinoma that *SETD5* depletion downregulated interferon-mediated inflammatory responses and reduced PKM expression, leading to decreased glycolysis activity [43]. The finding that *SETD5* loss compromises interferon signaling in liver cancer cells is of particular significance in the context of our immunogenomic results, as it provides a potential mechanistic link between *SETD5* expression levels and both the immune checkpoint signatures and the innate immune cell infiltration patterns such as macrophages and dendritic cells observed across tumor types. These complementary functional data suggest that *SETD5* may operate as a context-dependent epigenetic scaffolding protein whose deregulation is associated with transcriptional fidelity, metabolic programming, and immune signaling pathways.

We investigated the relationship between *SETD5* and the tumor immune microenvironment to explore mechanisms that may underlie its context-dependent prognostic associations. Our immunogenomic analysis revealed that elevated *SETD5* expression is preferentially associated with innate immune cell infiltration such as macrophages, neutrophils, and dendritic cells while associations with cytotoxic lymphocytes were variable and tumor-type dependent, being absent in ESCA and HNSC. Although correlations between *SETD5* and individual immune checkpoint genes were modest in magnitude (ρ = 0.11–0.39 across tumor types), strong inter-checkpoint co-expression was observed, particularly between *LAG3* and *PDCD1* (ρ = 0.84 in COAD; ρ = 0.78 in READ), *CTLA4* and *PDCD1* (ρ = 0.75 in COAD; ρ = 0.69 in READ), and *HAVCR2* and *LAG3* (ρ = 0.69 in COAD; ρ = 0.66 in READ). This pattern of coordinated checkpoint co-expression, rather than a direct *SETD5*–checkpoint relationship, is consistent with *SETD5* marking a tumor state in which T-cell exhaustion programs are broadly engaged. Exhausted T cells express high levels of inhibitory receptors including *PD-1*, *CTLA-4*, *LAG-3*, and *TIM-3*, which severely limit their proliferative and cytotoxic capacity [44,45]. Notably, co-expression of *TIM-3* with *PD-1* identifies the most severely exhausted CD8^+^ T cell subset, characterized by reduced ability to proliferate and secrete IL-2, TNF, and IFN-γ [44,46,47]. Meta-analyses have demonstrated that high *TIM-3* expression is an independent prognostic indicator for poor overall survival (HR = 1.54–1.67) across multiple cancer types [48,49], with *TIM-3* positivity correlating with worse progression-free survival in various malignancies [50]. Taken together, the predominance of innate immune cell associations including macrophages and neutrophils alongside the coordinated checkpoint co-expression observed in *SETD5*-high tumors, suggests an immune landscape shaped by innate immunosuppression rather than adaptive cytotoxic activity, with critical implications for immunotherapy responsiveness.

The immunosuppressive character of this microenvironment is further supported by the robust correlations between *SETD5* and innate immune populations, most notably macrophages, neutrophils, and dendritic cells alongside the coordinated checkpoint co-expression patterns described above. Tumor-associated macrophages (TAMs) create immunosuppressive niches through *PD-L1* expression and secretion of arginase 1, IL-10, and TGF-β [51,52], which inhibit CD8^+^ T cell function and promote T cell exhaustion [44,45]. Tumor-associated neutrophils (TANs) can adopt both anti-tumorigenic (N1) and pro-tumorigenic (N2) phenotypes, with the latter promoting angiogenesis, matrix remodeling, and immunosuppression through secretion of VEGF, MMP-9, and TGF-β [53], suggesting a potential role for *SETD5* in fostering a pro-tumorigenic innate immune niche. Similarly, tumor-associated dendritic cells with compromised antigen-presenting function promote immunosuppression through secretion of VEGF, TGF-β, and IL-10 [54,55]. While immune checkpoint inhibitors have demonstrated clinical efficacy in various solid tumors [56], resistance remains a significant challenge. Our analysis cannot determine whether *SETD5*-high tumors would respond to immunotherapy or exhibit resistance mechanisms, such as primary resistance in tumors with predominant innate immune infiltration and limited cytotoxic T-cell engagement (as observed in ESCA and HNSC); adaptive resistance with upregulation of alternative checkpoints (TIM-3, LAG-3, TIGIT) during treatment; or acquired resistance through mutations in antigen presentation or interferon-γ signaling pathways [4,57]. Given the limitations of bulk RNA-seq deconvolution analyses, future single-cell RNA sequencing and spatial transcriptomics studies will be important to validate these associations and define the cellular and spatial context of *SETD5* within the tumor microenvironment.

The convergence of our immunogenomic findings characterized by innate immune cell infiltration, modest *SETD5*–checkpoint correlations, and strong inter-checkpoint co-expression with the growing body of functional evidence on *SETD5* supports a model in which *SETD5* overexpression marks, and may contribute to, the establishment of an immunosuppressive tumor microenvironment through multiple, potentially interconnected mechanisms. *SETD5*-mediated regulation of interferon signaling pathways, as demonstrated in hepatocellular carcinoma [43], may directly influence the expression of immune checkpoint molecules and the polarization state of infiltrating innate immune cells, including macrophages and dendritic cells, the populations most consistently correlated with *SETD5* expression in our analysis. Concurrently, *SETD5*-driven metabolic reprogramming, particularly glycolysis enhancement through the EP300/HIF-1α axis [9] and PKM2 nuclear translocation [11], could create a lactate-rich microenvironment that further promotes macrophage polarization toward immunosuppressive phenotypes and neutrophil recruitment to the tumor microenvironment. Notably, the near-zero and slightly negative correlations between *SETD5* and checkpoint molecules observed in LUSC (*CTLA4*: ρ = −0.03; *PDCD1*: ρ = −0.02; *HAVCR2*: ρ = −0.08) underscore the tumor-type specificity of these relationships and caution against generalizing a single mechanistic model across all *SETD5*-overexpressing malignancies. This integrative view positions *SETD5* at the intersection of epigenetic, metabolic, and immune regulatory circuits, providing a rationale for combinatorial therapeutic strategies targeting *SETD5*-associated pathways in conjunction with immune checkpoint inhibitors, although these findings remain correlative and require experimental validation.

This study has limitations that should be acknowledged. First, although robust, our analyses are based on publicly available in silico data, and the correlative nature of the findings precludes causal inference. The positive correlations between *SETD5* expression and immune cell infiltration scores for macrophages, neutrophils, and dendritic cells derived from bulk RNA-seq deconvolution algorithms such as CIBERSORT, cannot distinguish between genuine immune cell presence and potential computational artifacts. Furthermore, the absence of detectable correlations for CD8^+^ T cells in ESCA and HNSC, and for B cells in most cohorts, may reflect biological tumor-type specificity or limitations of the deconvolution algorithm in resolving rare cell populations in these datasets. Second, the TCGA datasets used in this study lack matched normal tissue controls for several cancer types, which may limit the statistical power of methylation and expression comparisons, as observed for COAD and READ. Third, while we identified consistent positive correlations between *SETD5* and immune checkpoint genes most notably *CD274* and *HAVCR2* in gastrointestinal tumor types these associations are modest in magnitude and do not establish that *SETD5* directly regulates checkpoint expression or T-cell exhaustion. The observed correlation may reflect shared upstream regulatory mechanisms or co-regulation by third-party factors. Finally, we acknowledge that future studies using *SETD5* knockout or knockdown models, coupled with single-cell RNA sequencing of the tumor immune microenvironment, will be essential to delineate the causal relationships suggested by our integrative analysis.

In this study, we performed a comprehensive pan-cancer characterization of *SETD5* across nine malignancies by integrating transcriptomic, genomic, epigenetic, and immunogenomic data. *SETD5* was consistently overexpressed in tumor tissues compared with normal controls, predominantly independent of mutation burden. Instead, promoter hypomethylation and copy-number amplification emerged as potential drivers of *SETD5* deregulation, particularly in BLCA, LIHC, and ESCA. Functional enrichment analyses further associated *SETD5* with chromatin-related regulatory complexes involved in macromolecule methylation and transcriptional elongation, supporting its proposed role in epigenetic regulation. Immunogenomic analyses revealed that elevated *SETD5* expression correlates with increased infiltration of innate immune populations, including macrophages, neutrophils and dendritic cells across tumor types, with variable CD8^+^ T cell associations depending on tumor context. Additionally, *SETD5*-high tumors were characterized by strong coordinated co-expression among immune checkpoint genes, suggesting a tumor microenvironment permissive to immune evasion.

## 5. Conclusions

Collectively, our findings suggests a model in which *SETD5* may promote tumor progression through interconnected mechanisms involving epigenetic reprogramming, metabolic adaptation, and modulation of the tumor immune microenvironment, including association with coordinated checkpoint programs and immune cell exhaustion signatures. Although our analyses are primarily correlative, the integration of pan-cancer data with previously reported functional evidence highlights *SETD5* as a promising candidate for future mechanistic and translational studies.

## Figures and Tables

**Table 1:** Frequency of mutations and copy number alterations in 8 types of tumors.

Cancer Type	Mutation Frequency (%)	Amplification Frequency (%)	Homodel Frequency (%)
COAD	3.9	0.0	0.3
READ	4.7	0.0	0.6
LUAD	3.0	0.2	0.2
LUSC	5.9	0.2	0.8
LIHC	1.6	1.6	0.0
HNSC	1.2	0.6	1.0
ESCA	2.0	2.6	1.0
BLCA	0.7	7.5	0.0

## Data Availability

The data sets and methods used and/or analyzed in the current study are available within the manuscript. All data analyzed and materials used in this study are available from the corresponding author upon request.

## Supplementary Materials

The following supporting information can be downloaded at: https://www.mdpi.com/article/10.3390/cimb48070742/s1.

## Abbreviations

The following abbreviations are used in this manuscript:

SETD5	SET domain-containing protein 5
CNA	Copy number alteration
GISTIC	Genomic Identification of Significant Targets in Cancer
TCGA	The Cancer Genome Atlas
PPI	Protein–protein interaction
GO	Gene ontology
KEGG	Kyoto Encyclopedia of Genes and Genomes
TPM	Transcripts per million
AMP	Gene amplification
BLCA	Bladder urothelial carcinoma
COAD	Colon adenocarcinoma
ESCA	Esophageal carcinoma
HNSC	Head and neck squamous cell carcinoma
LIHC	Liver hepatocellular carcinoma
LUAD	Lung adenocarcinoma
LUSC	Lung squamous cell carcinoma
READ	Rectum adenocarcinoma
STAD	Stomach adenocarcinoma
TAM	Tumor-associated macrophage
TAN	Tumor-associated neutrophil

## References

[1] Hanahan D. (2022). Hallmarks of cancer: New dimensions. Cancer Discov..

[2] Flavahan W.A., Gaskell E., Bernstein B.E. (2017). Epigenetic plasticity and the hallmarks of cancer. Science.

[3] Peng D., Kryczek I., Nagarsheth N., Zhao L., Wei S., Wang W., Sun Y., Zhao E., Vatan L., Szeliga W. (2015). Epigenetic silencing of T_H_1-type chemokines shapes tumour immunity and immunotherapy. Nature.

[4] Sharma P., Hu-Lieskovan S., Wargo J.A., Ribas A. (2017). Primary, adaptive, and acquired resistance to cancer immunotherapy. Cell.

[5] Feinberg A.P., Koldobskiy M.A., Göndör A. (2016). Epigenetic modulators, modifiers and mediators in cancer a etiology and progression. Nat. Rev. Genet..

[6] Zhou X., Chen W., Zhuang D., Xu G., Puyang Y., Rui H. (2025). Knockdown of *SETD5* inhibits colorectal cancer cell growth and stemness by regulating PI3K/AKT/mTOR pathway. Biochem. Genet..

[7] Taneja L., Bhardwaj S., Yadav A.K. (2025). *SETD5* in glioma cells conferred TRAIL resistance induction. Sci. Rep..

[8] Abe M., Kubota N., Yamazaki K., Miura E., Hirabayashi K., Ishii M., Shirakawa H., Kikuta K., Murayama Y., Nakagawa R. (2025). High immunohistochemical expression of *SETD5* as a candidate pathological factor for dedifferentiation and prognosis in liposarcoma. Pathol. Int..

[9] Yang Z., Zhang C., Liu X., Che N., Feng Y., Xuan Y. (2022). *SETD5* regulates glycolysis in breast cancer stem-like cells and fuels tumor growth. Am. J. Pathol..

[10] Wang Z., Hausmann S., Lyu R., Li T.-M., Lofgren S.M., Flores N.M., Fuentes M.E., Caporicci M., Yang Z., Meiners M.J. (2020). *SETD5*-coordinated chromatin reprogramming regulates adaptive resistance to targeted pancreatic cancer therapy. Cancer Cell.

[11] Liu X., Cui Y., Gong J., Yu X., Cui Y., Xuan Y. (2025). *SETD5* facilitates stemness and represses ferroptosis via m6A-mediating PKM2 stabilization in non-small cell lung cancer. Oncogene.

[12] Li M., Hou Y., Zhang Z., Zhang B., Huang T., Sun A., Shao G., Lin Q. (2023). Structure, activity and function of the lysine methyltransferase SETD5. Front. Endocrinol..

[13] Jiang Z., Zhao J., Zou H., Cai K. (2022). CircRNA *PTPRM* promotes non-small cell lung cancer progression by modulating the miR-139-5p/SETD5 axis. Technol. Cancer Res. Treat..

[14] Poissonnier L., Villain G., Soncin F., Mattot V. (2014). miR126-5p repression of ALCAM and *SetD5* in endothelial cells regulates leucocyte adhesion and transmigration. Cardiovasc. Res..

[15] Yu G., Wang L.G., Han Y., He Q.Y. (2012). clusterProfiler: An R package for comparing biological themes among gene clusters. Omics.

[16] Lazaro-Camp V.J., Salari K., Meng X., Yang S. (2021). *SETDB1* in cancer: Overexpression and its therapeutic implications. Am. J. Cancer Res..

[17] Han T.-S., Kim D.-S., Son M.-Y., Cho H.-S. (2024). SMYD family in cancer: Epigenetic regulation and molecular mechanisms of cancer proliferation, metastasis, and drug resistance. Exp. Mol. Med..

[18] Vogelstein B., Papadopoulos N., Velculescu V.E., Zhou S., Diaz L.A., Kinzler K.W. (2013). Cancer genome landscapes. Science.

[19] McCabe M.T., Ott H.M., Ganji G., Korenchuk S., Thompson C., Van Aller G.S., Liu Y., Graves A.P., Iii A.D.P., Diaz E. (2012). *EZH2* inhibition as a therapeutic strategy for lymphoma with *EZH2*-activating mutations. Nature.

[20] Copeland R., Solomon M., Richon V. (2009). Protein methyltransferases as a target class for drug discovery. Nat. Rev. Drug Discov..

[21] De Angelis M.T., Rizzuto A., Amaddeo A., Sagnelli C., Vono N., Reda M., Lise V., Parrillo L., De Marco C., Malanga D. (2025). Distinctive chromosomal, mutational and transcriptional profiling in colon versus rectal cancers. J. Transl. Med..

[22] Zengin T., Önal-Süzek T. (2021). Comprehensive profiling of genomic and transcriptomic differences between risk groups of lung adenocarcinoma and lung squamous cell carcinoma. J. Pers. Med..

[23] Beroukhim R., Mermel C.H., Porter D., Wei G., Raychaudhuri S., Donovan J., Barretina J., Boehm J.S., Dobson J., Urashima M. (2010). The landscape of somatic copy-number alteration across human cancers. Nature.

[24] Zack T.I., Schumacher S.E., Carter S.L., Cherniack A.D., Saksena G., Tabak B., Lawrence M.S., Zhang C.-Z., Wala J., Mermel C.H. (2013). Pan-cancer patterns of somatic copy number alteration. Nat. Genet..

[25] Van Tongelen A., Loriot A., De Smet C. (2017). Oncogenic roles of DNA hypomethylation through the activation of cancer-germline genes. Cancer Lett..

[26] Smith J., Sen S., Weeks R.J., Eccles M.R., Chatterjee A. (2020). Promoter DNA hypermethylation and paradoxical gene activation. Trends Cancer.

[27] Zhu J., Liu Z., Liang X., Wang L., Wu D., Mao W., Shen D. (2022). A pan-cancer study of KMT2 family as therapeutic targets in cancer. J. Oncol..

[28] Bushara O., Wester J.R., Jacobsen D., Sun L., Weinberg S., Gao J., Jennings L.J., Wang L., Lauberth S.M., Yue F. (2023). Clinical and histopathologic characterization of *SETD2*-mutated colorectal cancer. Hum. Pathol..

[29] Zhong Y., Wang R., Huang Z., Hu Z., Peng B., Chen B., Sun L. (2025). Identification of *SETD4* as an onco-immunological biomarker encompassing the tumor microenvironment, prognoses, and therapeutic responses in various human cancers. Immun. Inflamm. Dis..

[30] Yang W.-Q., Xiong Q.-P., Ge J.-Y., Li H., Zhu W.-Y., Nie Y., Lin X., Lv D., Li J., Lin H. (2021). THUMPD3-TRMT112 is a m^2^G methyltransferase working on a broad range of tRNA substrates. Nucleic Acids Res..

[31] Wang C., Ulryck N., Herzel L., Pythoud N., Kleiber N., Guérineau V., Jactel V., Moritz C., Bohnsack M.T., Carapito C. (2023). *N*^2^-methylguanosine modifications on human tRNAs and snRNA U6 are important for cell proliferation, protein translation and pre-mRNA splicing. Nucleic Acids Res..

[32] Kuechler A., Zink A.M., Wieland T., Lüdecke H.-J., Cremer K., Salviati L., Magini P., Najafi K., Zweier C., Czeschik J.C. (2015). Loss-of-function variants of *SETD5* cause intellectual disability and the core phenotype of microdeletion 3p25.3 syndrome. Eur. J. Hum. Genet..

[33] Fan L., Sun W., Lyu Y., Ju F., Sun W., Chen J., Ma H., Yang S., Zhou X., Wu N. (2024). Chrom-seq identifies RNAs at chromatin marks. Sci. Adv..

[34] Sashiyama S., Nakagawa T., Nakagawa M., Hosogane M., Watanabe Y., Ashitomi H., Yamane K., Shibuya N., Moroishi T., Nakayama K. (2025). KBG syndrome-associated protein ANKRD11 regulates *SETD5* expression to modulate rRNA levels and translation. iScience.

[35] Black J.C., Van Rechem C., Whetstine J.R. (2012). Histone lysine methylation dynamics: Establishment, regulation, and biological impact. Mol. Cell..

[36] Greer E.L., Shi Y. (2012). Histone methylation: A dynamic mark in health, disease and inheritance. Nat. Rev. Genet..

[37] Kwak H., Lis J.T. (2013). Control of transcriptional elongation. Annu. Rev. Genet..

[38] Jonkers I., Lis J.T. (2015). Getting up to speed with transcription elongation by RNA polymerase II. Nat. Rev. Mol. Cell Biol..

[39] Sun L., Zhang H., Gao P. (2022). Metabolic reprogramming and epigenetic modifications on the path to cancer. Protein Cell.

[40] Shen R., Ruan H., Lin S., Liu B., Song H., Li L., Ma T. (2022). Lysine succinylation, the metabolic bridge between cancer and immunity. Genes Dis..

[41] Lu K.-Q., Li Z.-L., Zhang Q., Yin Q., Zhang Y.-L., Ni W.-J., Jiang L.-Z., He W., Wang B. (2024). *CDK12* is a potential biomarker for diagnosis, prognosis and immunomodulation in pan-cancer. Sci. Rep..

[42] Shi J., Yu L., Zhu C., Zhong H. (2023). Knockdown of *SETD5* inhibited glycolysis and tumor growth in gastric cancer cells by down-regulating Akt signaling pathway. Open Life Sci..

[43] Park M., Moon B., Kim J.-H., Park S.-J., Kim S.-K., Park K., Kim J., Kim S.-Y., Kim J.-H., Kim J.-A. (2022). Downregulation of *SETD5* suppresses the tumorigenicity of hepatocellular carcinoma cells. Mol. Cells.

[44] McLane L.M., Abdel-Hakeem M.S., Wherry E.J. (2019). CD8 T cell exhaustion during chronic viral infection and cancer. Annu. Rev. Immunol..

[45] Wherry E.J., Kurachi M. (2015). Molecular and cellular insights into T cell exhaustion. Nat. Rev. Immunol..

[46] Sakuishi K., Apetoh L., Sullivan J.M., Blazar B.R., Kuchroo V.K., Anderson A.C. (2010). Targeting Tim-3 and PD-1 pathways to reverse T cell exhaustion and restore anti-tumor immunity. J. Exp. Med..

[47] Fourcade J., Sun Z., Benallaoua M., Guillaume P., Luescher I.F., Sander C., Kirkwood J.M., Kuchroo V., Zarour H.M. (2010). Upregulation of *Tim-3* and *PD-1* expression is associated with tumor antigen-specific CD8^+^ T cell dysfunction in melanoma patients. J. Exp. Med..

[48] Zang K., Hui L., Wang M., Huang Y., Zhu X., Yao B. (2021). *TIM-3* as a prognostic marker and a potential immunotherapy target in human malignant tumors: A meta-analysis and bioinformatics validation. Front. Oncol..

[49] Xu W., Qi F., Jiao R., Zheng L., Zhang Y., Hou D., Liu Y., Kang Z. (2020). Prognostic and clinicopathological value of high expression of *TIM-3* in different cancer types: A meta-analysis. Precis. Med. Sci..

[50] Granier C., Dariane C., Combe P., Verkarre V., Urien S., Badoual C., Roussel H., Mandavit M., Ravel P., Sibony M. (2017). *Tim-3* expression on tumor-infiltrating PD-1^+^CD8^+^ T cells correlates with poor clinical outcome in renal cell carcinoma. Cancer Res..

[51] Kuang D.-M., Zhao Q., Peng C., Xu J., Zhang J.-P., Wu C., Zheng L. (2009). Activated monocytes in peritumoral stroma of hepatocellular carcinoma foster immune privilege and disease progression through *PD-L1*. J. Exp. Med..

[52] Pan Y., Yu Y., Wang X., Zhang T. (2020). Tumor-associated macrophages in tumor immunity. Front. Immunol..

[53] Jaillon S., Ponzetta A., Di Mitri D., Santoni A., Bonecchi R., Mantovani A. (2020). Neutrophil diversity and plasticity in tumour progression and therapy. Nat. Rev. Cancer.

[54] Scarlett U.K., Rutkowski M.R., Rauwerdink A.M., Fields J., Escovar-Fadul X., Baird J., Cubillos-Ruiz J.R., Jacobs A.C., Gonzalez J.L., Weaver J. (2012). Ovarian cancer progression is controlled by phenotypic changes in dendritic cells. J. Exp. Med..

[55] Veglia F., Gabrilovich D.I. (2017). Dendritic cells in cancer: The role revisited. Curr. Opin. Immunol..

[56] Haslam A., Prasad V. (2019). Estimation of the percentage of US patients with cancer who are eligible for and respond to checkpoint inhibitor immunotherapy drugs. JAMA Netw. Open.

[57] Koyama S., Akbay E.A., Li Y.Y., Herter-Sprie G.S., Buczkowski K.A., Richards W.G., Gandhi L., Redig A.J., Rodig S.J., Asahina H. (2016). Adaptive resistance to therapeutic PD-1 blockade is associated with upregulation of alternative immune checkpoints. Nat. Commun..

