# Supplementary material for: Integrative Analysis Uncovers SETD5 as an Epigenetic Regulator of Transcriptional and Immune Tumor Programs Across Human Cancers
Source: Curr Issues Mol Biol. 2026 Jul 21;48(7):742. doi: 10.3390/cimb48070742 (PMC13409287; doi:10.3390/cimb48070742)
Supplement: Supplementary file 1 [file cimb-48-00742-s001.zip › Supplementary_figures.pdf]

Supplementary figures

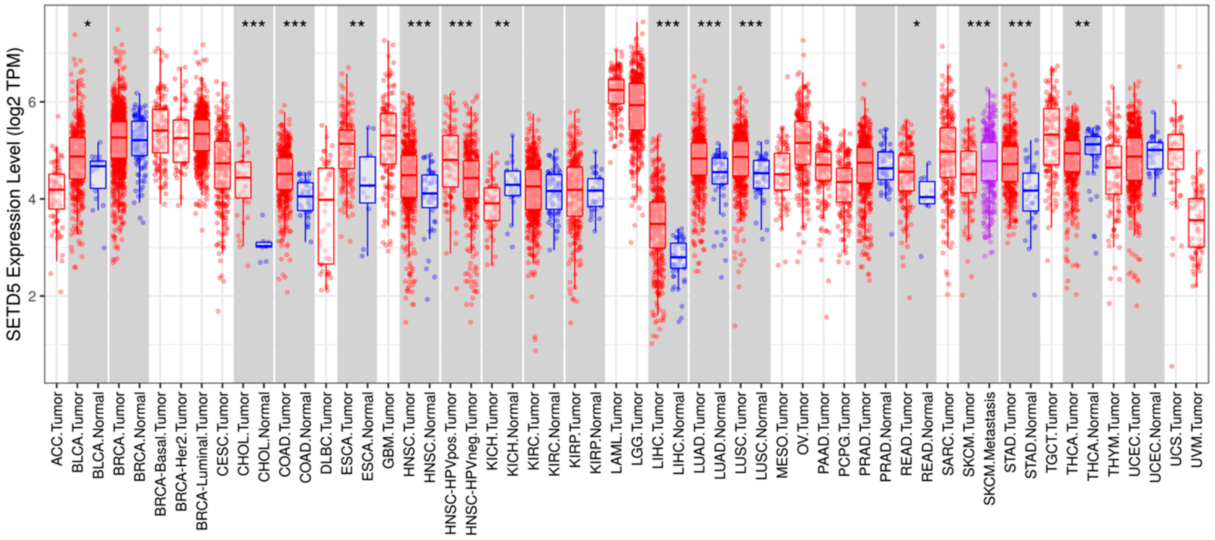

Supplementary Figure S1. mRNA expression between tumor and normal tissue. *SETD5* is highly expressed in several tumors. (Bladder urothelial carcinoma – BLCA; Colon adenocarcinoma – COAD; Esophageal carcinoma - ESCA; Head and neck squamous cell carcinoma – HNSC; Liver hepatocellular carcinoma – LIHC; Lung adenocarcinoma – LUAD; Lung squamous cell carcinoma – LUSC; Rectum adenocarcinoma – READ; Stomach adenocarcinoma - STAD). Wilcoxon test (*p<0.05, **p<0.01, ***p<0.001 and ****p<0.0001).

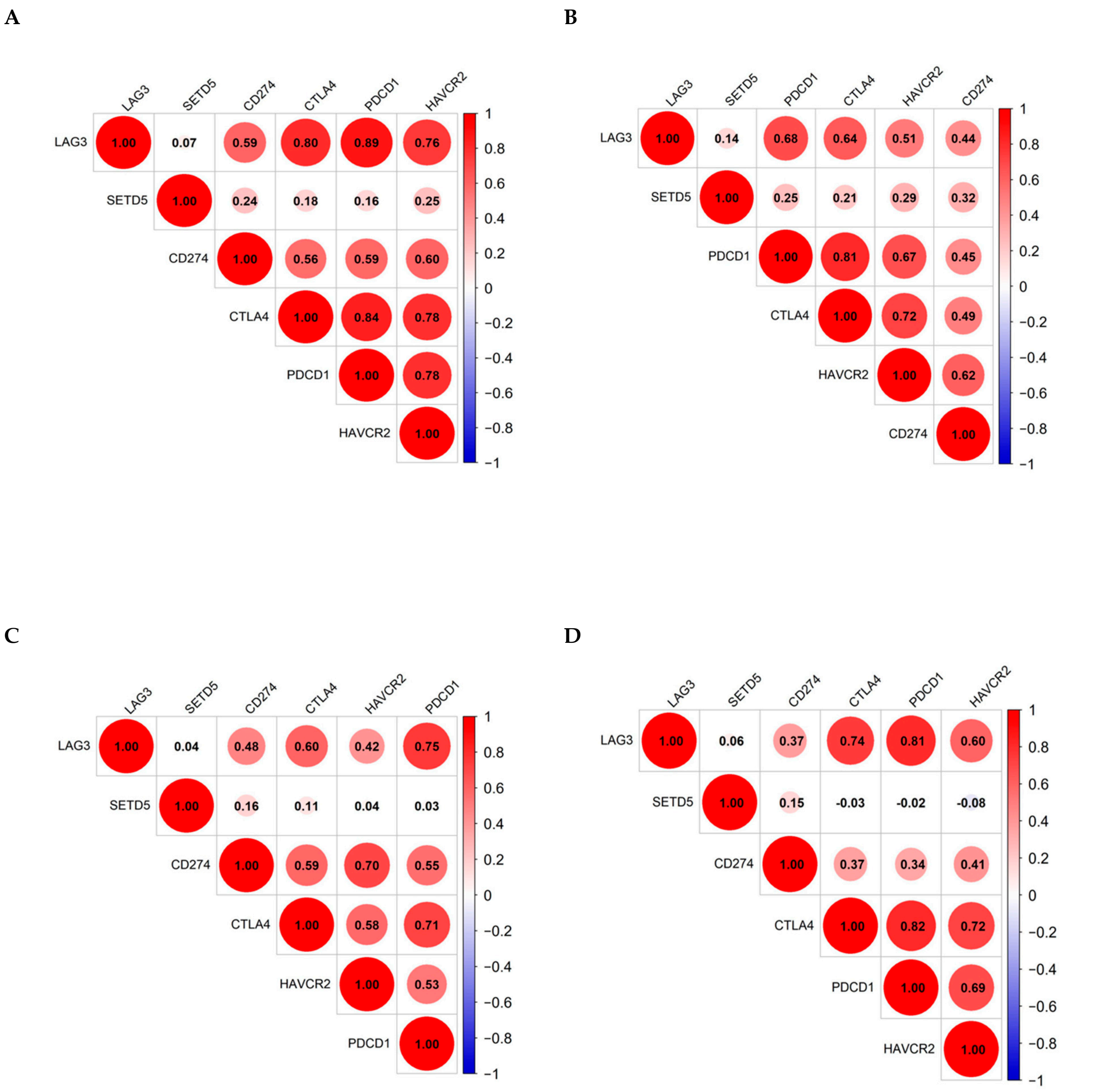

Supplementary Figure S2. Correlation analysis between *SETD5* and immune checkpoint genes across TCGA tumor samples. (A) HNSC. (B) LIHC. (C) LUAD. (D) LUSC Spearman correlation coefficients (ϱ) are represented by color intensity, ranging from negative (blue) to positive (red) correlations. Only pairwise complete observations were included in the analysis.

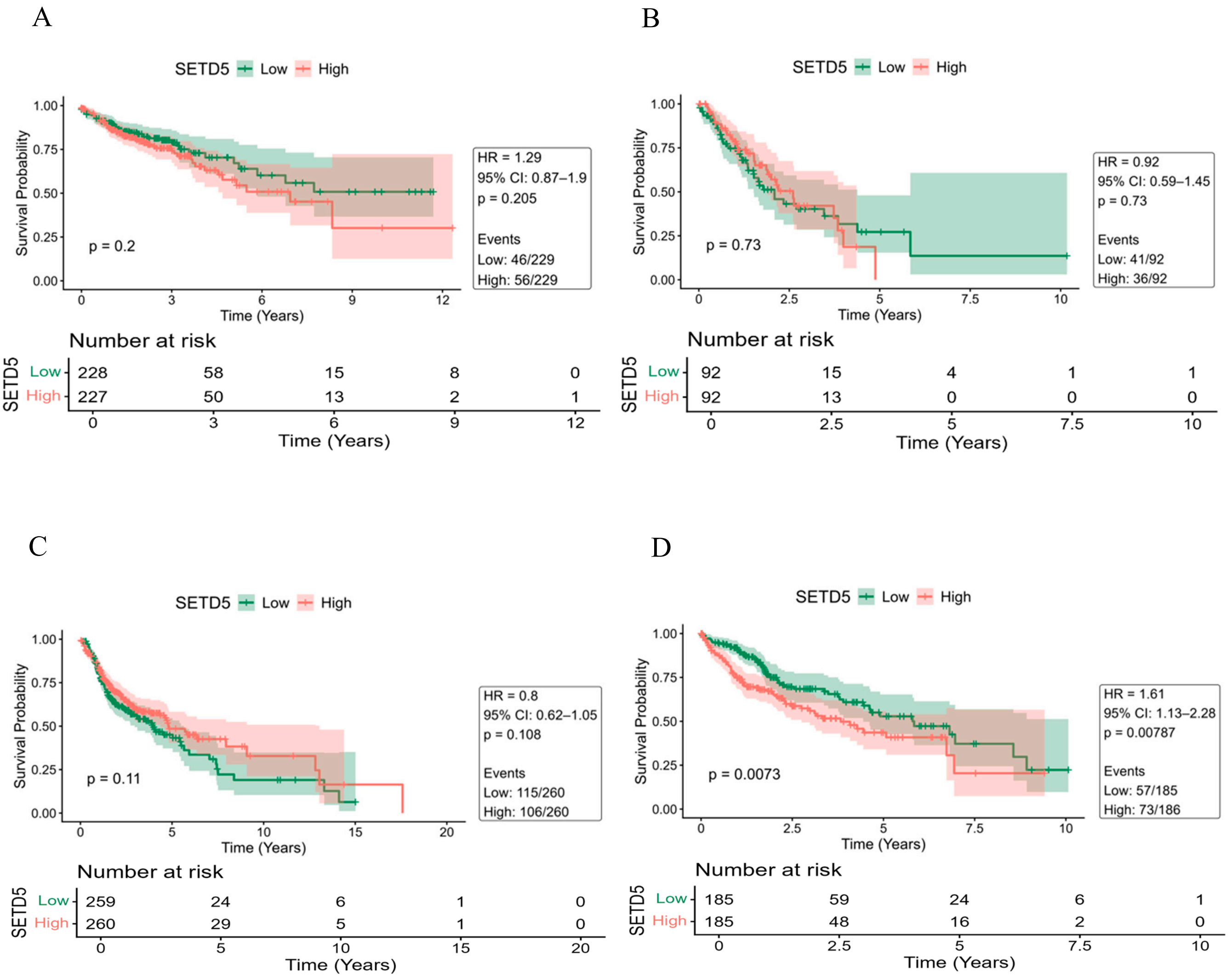
A
SETD5 Low High
Survival Probability
1.00
0.75
0.50
0.25
0.00
p = 0.2
HR = 1.29
95% CI: 0.87–1.9
p = 0.205
Events
Low: 46/229
High: 56/229
Time (Years)
Number at risk
SETD5
Low 228 58 15 8 0
High 227 50 13 2 1
0 3 6 9 12
Time (Years)
B
SETD5 Low High
Survival Probability
p = 0.73
HR = 0.92
95% CI: 0.59–1.45
p = 0.73
Events
Low: 41/92
High: 36/92
Time (Years)
Number at risk
SETD5
Low 92 15 4 1 1
High 92 13 0 0 0
0 2.5 5 7.5 10
Time (Years)
C
SETD5 Low High
Survival Probability
p = 0.11
HR = 0.8
95% CI: 0.62–1.05
p = 0.108
Events
Low: 115/260
High: 106/260
Time (Years)
Number at risk
SETD5
Low 259 24 6 1 0
High 260 29 5 1 0
0 5 10 15 20
Time (Years)
D
SETD5 Low High
Survival Probability
p = 0.0073
HR = 1.61
95% CI: 1.13–2.28
p = 0.00787
Events
Low: 57/185
High: 73/186
Time (Years)
Number at risk
SETD5
Low 185 59 24 6 1
High 185 48 16 2 0
0 2.5 5 7.5 10
Time (Years)

E

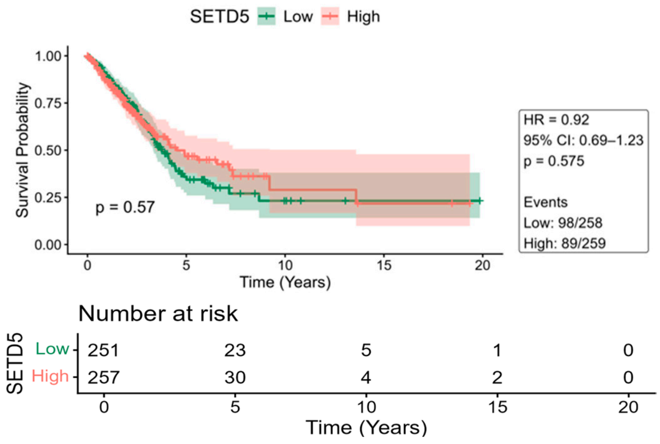

F

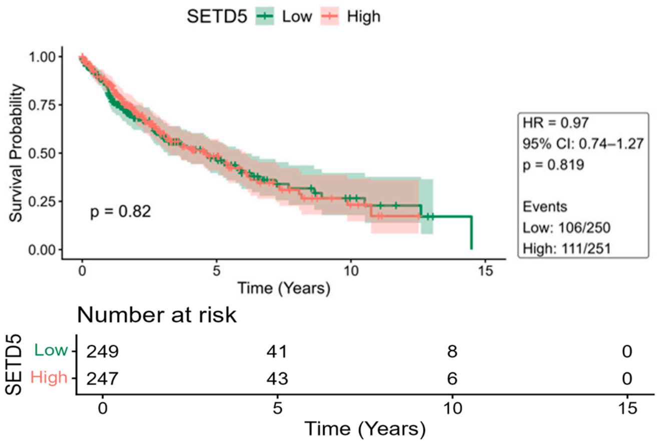

G

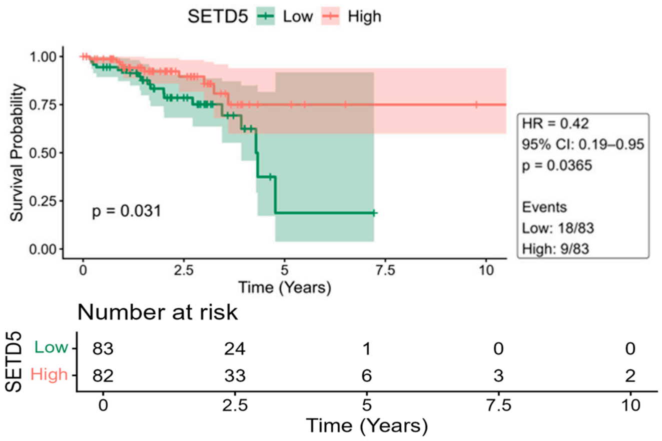

Supplementary Figure S3. Kaplan–Meier survival curves showing overall survival according to *SETD5* expression in 7 TCGA cancer patients. (A) COAD (B) ESCA (C) HNSC (D) LIHC (E) LUAD (F) LUSC (G) READ. Shaded areas represent the 95% confidence intervals. Tick marks indicate censored patients. The number of patients at risk at each time point is shown below the plot.
