# Supplementary material for: Integrative Analysis Uncovers SETD5 as an Epigenetic Regulator of Transcriptional and Immune Tumor Programs Across Human Cancers
Source: Curr Issues Mol Biol. 2026 Jul 21;48(7):742. doi: 10.3390/cimb48070742 (PMC13409287; doi:10.3390/cimb48070742)
Supplement: Supplementary file 1 [file cimb-48-00742-s001.zip › Supplementary_tables.pdf]

Supplementary tables

Supplementary Table S1: Sample size (n) of tumor and normal tissues included in the TIMER2.0 differential expression analysis corresponding to Figure S1.

TCGA Cohort	Cancer Type	Tumor samples (n)	Normal samples (n)
ACC	Adrenocortical carcinoma	79	—
BLCA	Bladder urothelial carcinoma	408	19
BRCA (Total)	Breast invasive carcinoma		112
BRCA-Basal	Basal subtype (triple-negative)	190	Shared BRCA normal samples
BRCA-HER2	HER2-enriched subtype	82	Shared BRCA normal samples
BRCA-Luminal	Luminal subtype (A and B)	564	Shared BRCA normal samples
CESC	Cervical squamous cell carcinoma and endocervical adenocarcinoma	304	3
CHOL	Cholangiocarcinoma	36	9
COAD	Colon adenocarcinoma	457	41
DLBC	Diffuse large B-cell lymphoma	48	—
ESCA	Esophageal carcinoma	184	11
GBM	Glioblastoma multiforme	163	—
HNSC (Total)	Head and neck squamous cell carcinoma	520	44
HNSC-HPVpos	Head and neck squamous cell carcinoma (HPV-positive)	74	Shared HNSC normal samples
HNSC-HPVneg	Head and neck squamous cell carcinoma (HPV-negative)	422	Shared HNSC normal samples
KICH	Kidney chromophobe	66	53
KIRC	Kidney renal clear cell carcinoma	533	72
KIRP	Kidney renal papillary cell carcinoma	290	32
LAML	Acute myeloid leukemia	173	—
LGG	Brain lower grade glioma	516	—
LIHC	Liver hepatocellular carcinoma	371	50
LUAD	Lung adenocarcinoma	515	59

LUSC	Lung squamous cell carcinoma	501	52
MESO	Mesothelioma	87	—
OV	Ovarian serous cystadenocarcinoma	303	—
PAAD	Pancreatic adenocarcinoma	178	4
PCPG	Pheochromocytoma and paraganglioma	179	3
PRAD	Prostate adenocarcinoma	497	52
READ	Rectum adenocarcinoma	166	10
SARC	Sarcoma	259	2
SKCM (Total)	Skin cutaneous melanoma	471	1
SKCM-Tumor	Primary skin cutaneous melanoma	103	—
SKCM-Metastasis	Metastatic skin cutaneous melanoma	368	—
STAD	Stomach adenocarcinoma	415	32
TGCT	Testicular germ cell tumors	150	—
THCA	Thyroid carcinoma	501	59
THYM	Thymoma	120	—
UCEC	Uterine corpus endometrial carcinoma	546	35
UCS	Uterine carcinosarcoma	57	—
UVM	Uveal melanoma	80	—

Abbreviations: ACC, adrenocortical carcinoma; BLCA, bladder urothelial carcinoma; BRCA, breast invasive carcinoma; CESC, cervical squamous cell carcinoma and endocervical adenocarcinoma; CHOL, cholangiocarcinoma; COAD, colon adenocarcinoma; DLBC, diffuse large B-cell lymphoma; ESCA, esophageal carcinoma; GBM, glioblastoma multiforme; HNSC, head and neck squamous cell carcinoma; KICH, kidney chromophobe; KIRC, kidney renal clear cell carcinoma; KIRP, kidney renal papillary cell carcinoma; LAML, acute myeloid leukemia; LGG, brain lower grade glioma; LIHC, liver hepatocellular carcinoma; LUAD, lung adenocarcinoma; LUSC, lung squamous cell carcinoma; MESO, mesothelioma; OV, ovarian serous cystadenocarcinoma; PAAD, pancreatic adenocarcinoma; PCPG, pheochromocytoma and paraganglioma; PRAD, prostate adenocarcinoma; READ, rectum adenocarcinoma; SARC, sarcoma; SKCM, skin cutaneous melanoma; STAD, stomach adenocarcinoma; TGCT, testicular germ cell tumors; THCA, thyroid carcinoma; THYM, thymoma; UCEC, uterine corpus endometrial carcinoma; UCS, uterine carcinosarcoma; UVM, uveal melanoma.
For BRCA molecular subtypes (Basal, HER2-enriched, and Luminal) and HNSC HPV subgroups (HPV-positive and HPV-negative), the same normal samples from the corresponding parent TCGA cohort were used for comparison.

Supplementary Table S2. Sample size (n) of normal and tumor tissue samples included in the UALCAN corresponding to Figure 1.

Cancer type	Normal tissue	Tumor tissue
BLCA	19	408
COAD	47	286
ESCA	11	184
HNSC	44	520
LIHC	50	371
LUAD	59	515
LUSC	52	503
READ	10	166
STAD	34	415

Supplementary Table S3: (A) Sample size (n) (B) Criteria inclusions and exclusions included in the cBIO Portal corresponding to Figure 2.

A

Cancer Type	Missense	Truncating	Inframe	Splice	Fusion	Total Mutation	Total patients
COAD	26	6	0	0	0	32	812
READ	4	1	0	0	0	5	107
LUSC	11	1	0	1	0	13	220
ESCA	9	0	0	0	0	9	453
LUAD	8	2	1	0	0	11	367
LIHC	NA	NA	NA	NA	NA	18	1124
HNSC	NA	NA	NA	NA	NA	8	641
BLCA	NA	NA	NA	NA	NA	5	686

B

Cancer type	Study ID	Total patients available (n)	Patients without mutation profiling (n)	Patients included in mutation analysis (n)	Criteria
COAD	coad_caseccc_2015<br>coad_cptac_2019<br>coad_cptac_gdc<br>coad_silu_2022<br>coad_tcga_gdc<br>crc_eo_2020	1831	1019	812	Patients classified as "Colon Adenocarcinoma" in the "Cancer Type Detailed" according to cBioPortal database
READ	bowel_colitis_msk_2022<br>coadread_mskresistance_2022<br>coadread_tcga	1130	1023	107	Patients classified as "Rectal Adenocarcinoma"

	coadread_tcga_pan_can_atlas_2018<br>crc_apc_impact_2020<br>crc_dd_2022<br>crc_eo_2020<br>crc_msk_2017<br>rectal_msk_2019<br>rectal_msk_2022<br>rectal_radiation_msk_2024				in the "Cancer Type Detailed" according to cBioPortal database
LUSC	bm_nsclc_mskcc_2023<br>luad_mskcc_2023_met_organotropism<br>lung_msk_mind_2020<br>lung_msk_pdx<br>lung_smc_2016<br>lusc_tcga<br>lusc_tcga_gdc<br>lusc_tcga_pan_can_atlas_2018<br>lusc_tcga_pub<br>nsclc_ctdx_msk_2022<br>nsclc_mskcc_2015<br>nsclc_pd1_msk_2018<br>nsclc_tracerx_2017	704	484	220	Patients classified as "Lung Squamous Cell Carcinoma" in the "Cancer Type Detailed" according to cBioPortal database
ESCA	egc_trap_msk_2020<br>esca_broad<br>esca_tcga<br>esca_tcga_gdc<br>esca_tcga_pan_can_atlas_2018<br>escc_icgc<br>escc_ucla_2014	941	488	453	Patients classified as "Esophageal Squamous Cell Carcinoma" in the "Cancer Type Detailed" according to cBioPortal database
LUAD	bm_nsclc_mskcc_2023<br>luad_cptac_gdc<br>luad_mskcc_2023_met_organotropism<br>luad_mskimpact_2021<br>luad_tcga<br>luad_tcga_gdc<br>lung_msk_mind_2020<br>lusc_cptac_2021<br>lusc_cptac_gdc<br>nsclc_mskcc_2015<br>nsclc_mskcc_2018	484	117	367	Patients classified as "Lung Adenocarcinoma" in the "Cancer Type Detailed" according to cBioPortal database
LIHC	hcc_clca_2024<br>hcc_inserm_fr_2015<br>hcc_jcopo_msk_2023<br>hcc_msk_2024<br>hcc_msk_venturaa_2018<br>hcc_mskimpact_2018<br>hcc_tcga_gdc<br>lihc_riken<br>lihc_tcga	2537	1413	1124	Patients classified as " Hepatocellular Carcinoma" in the "Cancer Type Detailed" according to cBioPortal database

	lihc_tcga_pan_can_atlas_2018				
HNSC	hnc_mskcc_2016<br>hnsc_broad<br>hnsc_jhu<br>hnsc_mdanderson_2013<br>hnsc_tcga<br>hnsc_tcga_pan_can_atlas_2018<br>hnsc_tcga_pub	702	61	641	Patients classified as "Head and Neck Cancer" in the "Cancer Type " according to cBioPortal database
BLCA	blca_bcan_hcrn_2022<br>blca_bgi<br>blca_cornell_2016<br>blca_dfarber_mskcc_2014<br>blca_msk_2024<br>blca_tcga	969	283	686	Patients classified as "Bladder cancer" in the "Cancer Type " according to cBioPortal database

Supplementary Table S4**.** Sample size (n) of normal and tumor tissue samples included in the TCGA corresponding to Figure 3.

Cancer type	Normal tissue	Tumor tissue
BLCA	21	418
BRCA	97	882
COAD	3	347
ESCA	16	185
LUAD	32	365
LUSC	42	282
LIHC	50	376
READ	3	95
STAD	2	199
HNCS	50	381

Supplementary Table S5. Sample size (n) of normal and tumor tissue samples included in the TCGA corresponding to Figures 6 and 7.

**Cohort-flow table**							
	**COAD**	**ESCA**	**HNSC**	**LIHC**	**LUAD**	**LUSC**	**READ**
**Total of TCGA samples retrieved**	481	184	520	371	540	511	166
**Duplicated samples**	23	0	0	0	23	10	0
**Number of final samples included in the study**	458	184	520	371	517	501	166

Supplementary Table S6. Protein interaction network of SETD5, including types of interactions and interaction scores obtained from the STRING database.

**Proteins**	**Type of interaction**	**Score**
THUMPD3	Coexpression and Textmining	0.732
KMT2E	Coexpression, Experiments, Databases, Textmining and Homology	0.702
CHD8	Coexpression, Experiments and Textmining	0.685
ANKRD11	Coexpression, Experiments and Textmining	0.669
GIGYF2	Coexpression and Textmining	0.617
SETD2	Coexpression, Textmining and Homology	0.599
AFF4	Coexpression, Experiments, Databases and Textmining	0.591
CDK12	Coexpression, Experiments and Databases	0.590
HUWE1	Coexpression, Experiments and Textmining	0.583
SETD4	Coexpression and Textmining	0.576
